# Untargeted Metabolite Profiling of Wild and *In Vitro* Propagated Sabah Jewel Orchid *Macodes limii* J.J. Wood & A.L. Lamb

**DOI:** 10.21315/tlsr2024.35.3.2

**Published:** 2024-10-07

**Authors:** Devina David, Nor Azizun Rusdi, Ruzaidi Azli Mohd Mokhtar, Lucky Poh Wah Goh, Jualang Azlan Gansau

**Affiliations:** 1 Faculty of Sustainable Agriculture, Universiti Malaysia Sabah, 90509 Sandakan, Sabah, Malaysia; 2 Institute of Tropical Biology and Conservation, Universiti Malaysia Sabah, 80400 Kota Kinabalu, Sabah, Malaysia; 3 Biotechnology Research Institute, Universiti Malaysia Sabah, 88400 Kota Kinabalu, Sabah, Malaysia; 4 Faculty of Science and Natural Resources, Universiti Malaysia Sabah, 88400 Kota Kinabalu, Sabah, Malaysia

**Keywords:** Orchidaceae, GC-MS, LC-MS/MS, DPPH Assay, Metabolomics, Orchidaceae, GC-MS, LC-MS/MS, Cerakin DPPH, Metabolomik

## Abstract

*Macodes limii* J.J. Wood & A.L. Lamb is a terrestrial jewel orchid native to Sabah, recognised for its sparkling golden-yellow venations, uniformly distributed on its leaves. Despite its high ornamental value, the exploration of the plant’s medicinal potential remains ambiguous. The current study was conducted to gain a fundamental understanding of the metabolite composition and regulation in *M. limii* plants from two different growing environments: wild and *in vitro* cultivation, as well as to analyse their phytochemical contents and antioxidant activity. The metabolite profiling of the *M . limii* plant extracts through gas chromatography-mass spectrometry (GC-MS) and liquid chromatography-tandem mass spectrometry (LC-MS/MS) analysis has tentatively identified compounds from various classes including sugars, carbohydrates, sugar alcohols, amino acids, organic acids, phenolic derivatives and lipid and lipid-like compounds. Subsequently, the multivariate statistical analysis confirmed the existence of significant metabolite variations across distinct growth environments. Notably, the leaf extract derived from wild-grown plants displayed the highest levels of total phenolic and flavonoid content, contributing significantly to its higher antioxidant activity as measured by the 2,2-diphenyl-1-picrylhydrazyl (DPPH) assay. The discovery has offered a fundamental understanding of the metabolites in *M. limii* jewel orchids, indicating that *in vitro* regenerated plants may represent a viable alternative for further investigating their therapeutic potential, thus helping to alleviate the impact on wild populations.

## Abstract (fr)

**Abstrak:**

*Macodes limii* J.J. Wood & A.L. Lamb merupakan orkid jewel daratan yang berasal dari Sabah, terkenal dengan urat pada daunnya yang berwarna kuning keemasan berkilauan. Walaupun ia mempunyai nilai hiasan yang tinggi, namun, kajian terhadap potensi tumbuhan ini dalam perubatan masih terhad. Kajian ini dijalankan untuk mendapatkan pemahaman asas mengenai komposisi dan regulasi metabolit dalam tumbuhan *M. limii* daripada dua persekitaran pertumbuhan yang berbeza: pertumbuhan liar dan penanam secara *in vitro*, serta menganalisis kandungan fitokimia dan aktiviti antioksidan tumbuhan ini. Pemprofilan metabolit esktrak tumbuhan *M. limii* melalui analisis kromatografi gas-spektrometri jisim (GC-MS) dan kromatografi cecair-spektrometri jisim tandem (LC-MS/MS) secara tentatif telah mengenal pasti sebatian daripada pelbagai kelas termasuk gula, karbohidrat, gula alkohol, asid amino, asid organik, derivatif fenolik dan sebatian lipid. Seterusnya, analisis statistik multivariat mengesahkan kewujudan variasi metabolit yang ketara pada persekitaran pertumbuhan yang berbeza. Terutamanya, ekstrak daun yang diambil daripada persekitaran liar menunjukkan paras kandungan fenolik dan flavonoid yang tertinggi, seterusnya menyumbang secara signifikan kepada aktiviti antioksidannya melalui cerakin 2,2-diphenyl-1-picrylhydrazyl (DPPH). Penemuan ini telah memberikan pengetahuan asas mengenai metabolit dalam orkid jewel *M. limii*, di mana ia menunjukkan bahawa tumbuhan yang dipropagasi semula melalui kaedah *in vitro* berpotensi menjadi alternatif dalam kajian teraputik pada masa akan datang, sekali gus dapat membantu mengurangkan kesan ke atas populasi liar.

**Highlights**
- The leaf extract of wild-grown *M. limii* exhibited the highest total phenolic content (TPC) and total flavonoid content (TFC), which significantly contributed to its high antioxidant activity.
- A phenolic glycoside compound, tentatively identified as kinsenoside (C_10_H_6_O_8_), was detected in leaf and root extracts of *M. limii* plants, which have previously been reported as the main active compound responsible for promoting certain jewel orchids as the “King of Medicine”.
- The potential of *in vitro* regenerated *M. limii* as alternative plants in future drug discovery research in mitigating the threats to the wild orchid population.

## INTRODUCTION

The Orchidaceae family ranks among the largest and most diverse families of flowering plants, comprising more than 28,000 species distributed across 736 genera (Christenhusz & Byng 2016). Orchids are usually prized for their exquisite flowers and long floral lifespan, while some orchids are appreciated for their velvety and distinctive foliage venations, which are also known as “jewel orchids”. *Anoectochilus*, *Goodyera*, *Ludisia* and *Macodes* are the four main genera of jewel orchids widely distributed in tropical Asia (Besi, Nikong, Pungga *et al*. 2020; Smidt *et al*. 2021). These terrestrial orchids have been recognised for their extraordinary therapeutic effects in protecting against liver injury (Yu *et al*. 2021), anti-oxidative and ageing-modulating activity (Wang *et al*. 2020), anti-hypoxia (Wu *et al*. 2021), and preventing cancer (Chac *et al*. 2021). Owing to their ornamental and medicinal attributes, many wild orchids are at risk of becoming endangered, primarily due to habitat destruction resulting from illegal collection, land clearance for development purposes, and the impact of climate change (Besi, Nikong, Justine *et al*. 2020; Juiling *et al*. 2020; Besi *et al*. 2021). Currently, the Orchidaceae has been listed in Appendices I and II of The Convention on International Trade in Endangered Species of Wild Fauna and Flora (CITES), therefore, efforts to mass propagate as well as to conserve them are significant. Fortunately, plant tissue culture has provided alternative solutions to tackle this issue by offering a relevant technique for the mass propagation of many wild orchid species including jewel orchids such as *Anoectochilus elatus* (Sherif *et al*. 2016), *A. roxburghii* (Wang *et al*. 2022), *A. formosanus* (Ket *et al*. 2004), *Ludisia discolor* (Burkhan *et al*. 2022) and *Macodes limii* (David *et al*. 2022).

In Sabah, the *Macodes limii* J.J. Wood & A.L. Lamb can be found in hill forests and lower montane ridge forests in Kota Belud to Mt. Kinabalu area, which is restricted to ultramafic soil (Wood *et al*. 2011). This endemic species has high ornamental potential, however, information on their biochemical compositions is still limited. According to previous studies, the medicinal properties of jewel orchids were attributed to the presence of various bioactive compounds such as flavonoids, polysaccharides, kinsenoside, organic acids, amino acids and other metabolites (Wu *et al*. 2020; Ye *et al*. 2020). To minimise the impact on natural plant populations, the implementation of plant tissue culture in jewel orchids regeneration including *A. formosanus* (Giap *et al*. 2018), *A. roxburghii* (Chung *et al*. 2021), and *A. elatus* (Shi *et al*. 2023) has served as viable alternatives to the production of essential bioactive compounds for medicinal uses.

Therefore, considering the potential of this indigenous jewel orchid, the current study was carried out to decipher the metabolites composition in *M. limii* plants by using gas chromatography (GC) and liquid chromatography (LC) coupled with mass spectrometry (MS). Mass spectrometry-based metabolite profiling has been long utilised to understand the physiology and biochemistry of plants including species and cultivar identification (Hamany *et al*. 2021), growth and development (Qin *et al*. 2020), response to external stress (Wong *et al*. 2020), nutritional requirements (Abdalla *et al*. 2021), as well as for natural product discovery (Carvalho *et al*. 2021). Further understanding of the correlation between phytochemical contents and their antioxidant activity in both wild and *in vitro* regenerated plants was also determined from this study. These findings are the first and may be useful for future exploration into the potential bioactive compounds derived from this plant.

## MATERIALS AND METHODS

### Plant Material

Wild-grown plants of *M. limii* were collected from Kota Belud-Ranau area, at a latitude of N5°87′13″ and longitude of E116°25′02″ which is situated along Kota Belud-Ranau at an elevation of 650 m–700 m on the Mt. Kinabalu range. The plants were brought back to the Institute of Tropical Biology and Conservation, Universiti Malaysia Sabah (UMS), and maintained under greenhouse conditions. The species was identified by Mr Jamirus Miun from the Forest Research Centre (FRC, Sandakan) prior deposited at the Sandakan Herbarium (SAN) of Forestry Research Centre, Sandakan, Sabah (specimen no: UMSDD-001-005/2018). Meanwhile, the *in vitro* propagated *M. limii* was established according to the protocol by David *et al*. (2022). The *in vitro* grown plants were previously cultured on half-Murashige & Skoog (Murashige & Skoog 1962) basal media (Sigma-Aldrich, MO, US) containing 3% (w/v) of sucrose (Sigma-Aldrich, MO, US), 0.1% (w/v) of activated charcoal (Sigma-Aldrich, MO, US), and 0.3% (w/v) Gelrite^TM^ (Duchefa Biochemie, Haarlem, Netherlands) and maintained under 12 h of photoperiod at 25 ± 2°C culture conditions.

### Phytochemical Analysis and Antioxidant Activity

#### Plant extraction

Fresh leaves and roots of the wild and *in vitro* regenerated plants were rapidly frozen with liquid nitrogen, followed by lyophilisation and grinding into a fine powder. Sample extraction was conducted according to Zain and Omar (2018) with slight modifications. To prepare the crude extract, 50 mg of dried powder from each sample was homogenised in 2 mL of pure methanol (Emsure, Analysis grade, Merck, Darmstadt, Germany). The homogenates were vortexed for 5 min and sonicated at 40°C for 30 min prior to centrifuging at 13,000 rpm at room temperature for 5 min. The supernatant for every sample was collected and kept at −20°C for further analysis.

#### Phytochemical tests for total flavonoids and phenolics

The total phenolic content (TPC) of tested samples was determined by Folin-Ciocalteu Reagent assay using the spectrophotometric method described by Sembiring *et al*. (2018). The methanolic extract (25 μL) of 1 mg/mL from leaf and root extracts was mixed with 100 μL of 10% (v/v) Folin-Ciocalteu reagent (Sigma-Aldrich, MO, US) and allowed to react for 5 min. After that, 75 μL of Na_2_CO_3_ (Sigma-Aldrich, MO, US) of 700 mM was added and the mixture was shaken well and incubated in dark conditions for 2 h. After incubation, the absorbance at 765 nm was measured with a spectrophotometric microplate reader (Multiskan Sky, Thermo Fisher Scientific, US). Gallic acid (Sigma-Aldrich, MO, US) was used as a standard at 0 μg/mL–100 μg/mL to produce a calibration curve. The total phenolics content in the plant extracts was expressed as mg of gallic acid equivalent (GAE)/g of dry extract.

Total flavonoid content (TFC) was quantified using the spectrophotometric method described by Chatatikun and Chiabchalard (2013) with some modifications. Plant extract (1 mg/mL, 50 μL) was mixed with 10 μL of 10% (w/v) aluminium chloride (AlCl_3_) (Sigma-Aldrich, MO, US), 150 μL of methanol, and 10 μL of 1 M of potassium acetate (CH_3_CO_2_K) (Sigma-Aldrich, MO, US), then vortexed for 5 min. The mixture was allowed to stand for 30 min at room temperature and the absorbance was measured at 415 nm with a spectrophotometric microplate reader. Quercetin (Sigma-Aldrich, MO, US) was used as standard at 0 μg/mL–100 μg/mL. The TFC was expressed as mg of quercetin equivalent (QE)/g of dry extract.

#### Antioxidant test by DPPH assay

The antioxidant activity of all extracts was evaluated through a free radical scavenging effect on 2,2-diphenyl-1-picrylhydrazyl. (DPPH) radical according to Chan *et al*. (2012) with slight modifications. For the reaction, 50 μL of methanolic extract was added to 200 μL of DPPH methanolic solution (0.1 mM) in a 96-well microplate. The mixtures were gently swirled for 1 min, covered, and allowed to react in the dark for 30 min at room temperature. Finally, the absorbance at 517 nm was measured using a spectrophotometric microplate reader. Ascorbic acid (Sigma-Aldrich, MO, US) was used as standard. Extracts were first tested at a single concentration of 10 mg/mL, and those showing good evidence of antioxidant activity were tested over a range of concentrations. The scavenging activity was determined as the percentage of inhibition using the following equation:


$$\%\;\text{scavenging activity}=(({\text{A}}_{\text{control}}-{\text{A}}_{\text{sample}})/{\text{A}}_{\text{control}})\times 100$$

where A_control_ is the absorbance of the control (DPPH solution without any sample) and A_sample_ is the absorbance of the test sample. The inhibition concentration (IC_50_) of the crude methanol extract was calculated by plotting the DPPH radical scavenging (%) against the concentration of the sample. IC_50_ is the concentration of the methanol extract (mg) required for scavenging DPPH radicals by 50%.

#### Statistical analysis

The analytical determinations were carried out in triplicate for each sample, and the experiments were repeated twice. Results were reported as mean ± standard deviation. Significant differences among the treatments were determined by analysis of variance (ANOVA) followed by Tukey HSD tests, where a *p*-value of less than 0.05 was regarded as significantly different using Statistical Package for the Social Sciences (SPSS) version 28.0 (IBM Corp., Armonk, NY, US) software. To interpret the relationships between antioxidant activity and phytochemical contents, a two-tailed Pearson’s correlation coefficient analysis was conducted using SPSS.

### Mass Spectrometry-based Metabolite Profiling Analysis

#### Metabolite extraction

The metabolite extraction for gas chromatography-mass spectrometry (GC-MS) and liquid chromatography-tandem mass spectrometry (LC-MS/MS) analysis was performed using a protocol described by Degu *et al*. (2014) with slight modifications. Lyophilised samples (30 mg) were extracted in a 1.5 mL mixture of methanol: chloroform: ultrapure water (2.5:1.0:1.0). All extraction solvents used for the metabolite extraction were purchased from Thermo Fisher Scientific (Geel, Belgium). Samples were vortexed for 30 s and ultrasonicated at 40°C for 30 min. Samples were then centrifuged at 12,000 rpm for 5 min, and the supernatant was transferred to a new vial prior addition of 300 μL chloroform and 300 μL of ultrapure water. Samples were later centrifuged at 12,000 rpm for 5 min, and 100 μL of the water/methanol phase was transferred to a vial dried in a vacuum concentrator (Eppendorf Concentrator Plus, Hamburg, Germany) at room temperature, prior to GC-MS analysis. The remaining water/methanol phase was transferred to ultra-performance liquid chromatography (UPLC) vials for LC-MS/MS analysis.

#### GC-MS analysis

To increase detection sensitivity, chemical derivatisation of the sample extract was performed according to the method described by Yang *et al*. (2018). This involved the addition of 80 μL of methoxyamine hydrochloride (Merck, Darmstadt Germany) (15 mg/mL in pyridine) at 37°C for 90 min, followed by the addition of 80 μL of N, O-bis(trimethylsilyl)trifluoroacetamide (BSFTA) (Sigma-Aldrich, MO, US) [BSFTA + 1% trimethylchlorosilane (TMCS)] for 60 min at 70°C. One microlitre (1 μL) of a derivatised sample was then injected with a split less mode into a GC-MS apparatus (Shimadzu GCMS-QP2020 NX, Shimadzu, Kyoto Japan). Helium was used as a carrier gas at a constant linear velocity of 36 cm/s. RTx-5 Sil MS column (30 m × 0.25 mm id × 0.25 film thickness) was used for separating each compound. The operating conditions of the column were as follows: The oven temperature program was maintained at 60°C for 5 min, then increased to 180°C at 6°C/min held for 8 min, and finally ramped to 300°C at 8°C/min and held for 5 min. The injector temperature was maintained at 280°C, pressure 75 kPa, total flow 28.9 mL/min, column flow 1.23 mL/min, linear velocity 40.5 cm/s and purge flow 3.0 mL/min. Mass spectra were collected in the electron ionisation (EI) mode with 70 eV ionisation energy in 40 to 600 m/z of scan range. The ion source temperature was adjusted to 200°C with 2.00 min of solvent cut time. Tuning was performed at the start of a run to ensure optimal instrument response and accurate mass-to-charge ratio and ion abundance measurement across the mass range of the instrument.

#### Metabolite profiling by LC-QTOF-MS

For liquid chromatography-quadrupole time-of-flight-mass spectrometry (LC-QTOF-MS) analysis, 1 μL of the extract was injected into a Vanquish UHPLC system (Thermo Scientific, Waltham, MA, US) coupled to the ultra-high resolution Qq-time-of-flight (TOF) Impact II mass spectrometer (Bruker Daltonics, GmbH, Bremen, Germany). The protocol for LC-MS/MS analysis was according to Lee *et al*. (2013) with slight modification. The separation was achieved using reverse phase Thermo Scientific™ Acclaim™ 120 C_18_, 2.2 μm (2.1 × 100 mm) column (MA, US). The column temperature was set at 40°C. The mobile phase consisted of A (0.1% of formic acid in water) and B (0.1% of formic acid in acetonitrile) (Thermo Fischer Scientific, MA, US) at a flow rate of 0.3 mL/min. Elution was programmed as a flow gradient that started with 1% of B held for 2 min; then increased to 99% B in 17 min and held for 3 min, decreasing from 99% to 2% in 1 min, and then maintained at 2% B for 5 min. For MS spectra acquisition, the instrument was set to capture features from 50 m/z–1,500 m/z in positive ion mode at 1.0 spectra/sec scan rate. Mass spectra were generated in the positive ion mode with an electrospray ionization (ESI) source. The MS data acquisition was performed from 50 m/z–1,500 m/z. The source gas temperature was set at 350°C with a flow of 8 L/min. A mass calibrant, sodium formate (Supelco, Darmstadt, Germany), was introduced between 0.1 min and 0.3 min during each acquisition. Post-acquisition analyte m/z values were calibrated against the introduced sodium formate.

#### Data processing

The raw data of all chromatogram peaks (.gqd) were converted to common data format (mzXML) files using a GC-MS Post-run Analysis by GC-MS Lab Solution in the Shimadzu system. Data processing was then conducted using a free software tool MZmine version 2.5.3 (http://mzmine.github.io/) (Pluskal *et al*. 2010), in combination with the automated data analysis pipeline (ADAP) GC3.0 deconvolution method by Ni *et al*. (2016). The identification of compounds for GC-MS data was performed by comparing their mass spectra with data from NIST 17 (NIST Mass Spectral Database, 2017 from National Institute of Standards and Technology, Gaithersburg, MD, US). Compound with ≥80% of similarity index according to the NIST 17 library was accepted and annotated. Meanwhile, for LC-MS data, the Compass DataAnalysis software (Bruker Daltonics, GmbH, Bremen, Germany) was used to convert all the raw spectral files (.d format) to centroided, lock-mass corrected format (.mzXML) for downstream analyses. The converted spectral files were uploaded to MZmine2 (v.2.5.3), and peak detection, deconvolution, isotope grouping, and alignment were conducted according to Du *et al*. (2020). A table with ion intensities for each feature was then exported (.csv format) for statistical analyses and the “Export for SIRIUS” module was used to generate an .mgf file for batch analysis with SIRIUS 4 (https://bio.informatik.uni-jena.de/sirius/) (Lehrstuhl Bioinformatik, Jena, Germany) (Dührkop *et al*. 2019; 2021) integrated with CSI:FingerID and CANOPUS. The SIRIUS 4 (v.4.0.1) was employed [using the default settings for a quadrupole-TOF (Q-TOF) instrument] to predict molecular formulas for unknown features and develop fragmentation trees for manual annotation of MS_2_ spectra. CSI:FingerID was then utilised to predict molecular properties of unknown features, which were then queried against molecular properties predicted for compounds in all available molecular databases. This in silico tool led to a ranked list of predicted structures, even when published MS_2_ spectra were not available for these structures. Next, the CANOPUS was deployed to classify features into molecular families using ClassyFire, providing biological insight in the absence of structural annotations (Djoumbou Feunang *et al*. 2016). Human Metabolome Database (http://www.hmdb.ca/), MassBank (http://www.massbank.jp), and METLIN (https://metlin.scripps.edu/) with an accuracy error of <5 ppm was also employed to verify the putatively annotated compounds.

#### Multivariate analysis

The CSV-format file was uploaded to the MetaboAnalyst 5.0 server (https://www.metaboanalyst.ca/) for successive analysis. To improve data quality for performing downstream statistical analysis, the data quality was checked, and sample normalisation was performed by log transformation and auto-scaling prior to multivariate analysis. Unsupervised principal component analysis (PCA) was conducted to visualise the grouping patterns of the *in vitro* cultures at different developmental stages. To maximise the differences and to detect those differences in metabolic profiling among groups, partial least squares discriminant analysis (PLS-DA) was then applied. Metabolites with variable importance in projection (VIP) score of >1.0 were identified as important metabolites for analysis of species-specific variation. The quality of the PCA and PLS-DA models was described by the cross-validation parameters, goodness-of-fit (R^2^) and goodness-of-prediction (Q^2^), representing the explained variance and the predictive capability of the model, respectively. Identification of compounds was performed by comparing their mass spectra with data from NIST 17. The mass spectral match factor for identification was set at 80 to reduce false positives.

## RESULTS

**Figure 1:**
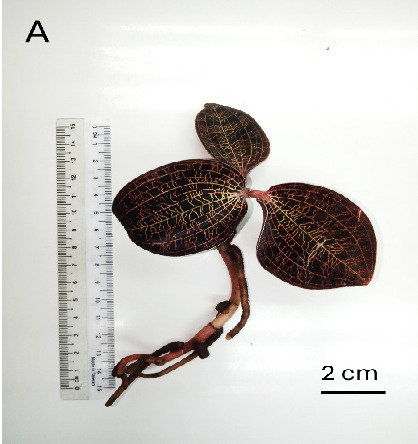

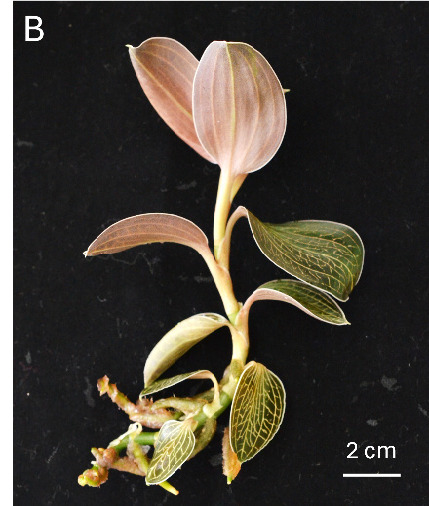
*Macodes limii* (a) wild-grown plant and (b) *in vitro* regenerated plant. Phytochemical analysis and antioxidant activity assay of wild and *in vitro* regenerated *M. limii*: (c) TPC; (d) TFC; (e) DPPH activity; and (f) IC50 DPPH (mg/mL). *Notes*: LW = leaf of wild plant; LV: leaf of *in vitro* regenerated plant; RW: root of wild plant; RV: root of *in vitro* regenerated plant. Means followed by the same lowercase letter did not differ significantly among treatments (Tukey’s test at 5%). The standard deviation is shown by error bars.

Plant extracts obtained from the leaf (L) and root (R) of the wild (W) [Fig. 1(a)] and *in vitro* regenerated (V) [Fig. 1(b)] *M. limii* were analysed for their phytochemical contents, antioxidant activity, as well as their metabolite profiles via GC-MS analysis.

### Phytochemical Contents

Using the gallic acid standard plot (y = 0.005 + 0.0187, R^2^ = 0.998), TPC in different extracts, ranged between 8.96 to 12.99 mg GAE/g of dry extract. LW extract yielded the highest TPC content with 12.99 ± 0.7 mg GAE/g of dry extract [Fig. 1(c)]. Meanwhile, the TFC of *M. limii* extracts ranged from 0.62 to 2.98 mg QE/g of dry extract, determined by the quercetin standard plot (y = 0.0072 + 0.0019, R^2^ = 0.998). TFC was significantly higher in LW than LV with 2.98 ± 0.24 mg QE/g dry extract and 2.47 ± 0.21 mg QE/g dry extract, respectively [Fig. 1(d)]. Root extracts from both sources exhibited the lowest levels of both TPC and TFC.

### Antioxidant Activity by DPPH Assay

The DPPH radical scavenging method was used to determine the antioxidant capacity of *M. limii* methanolic extracts. Ascorbic acid (0 μg/mL–100 μg/mL) was used as a positive control in this test. The greatest ability to scavenge DPPH radicals was observed in LW extract with 73% of scavenging activity, followed by LV (67%), RW (53%) and RV (49%) [Fig. 1(e)], with the concentrations to inhibit 50% of DPPH radicals (IC_50_) were at 6.22 ± 0.25 mg/mL, followed by 7.78 ± 0.07 mg/mL, 8.91 ± 0.57 mg/mL, and 10.04 ± 0.36 mg/mL, respectively [Fig. 1(f)]. To understand the contribution of phytochemicals (TPC and TFC) to the antioxidant potential of the plant extracts, a correlation was determined by Pearson’s correlation test. The result revealed a significant correlation (*p* < 0.01) between antioxidant activity by DPPH scavenging activity against TPC (r = 0.816) and TFC (r = 0.916). This finding explained that the antioxidant activity increased together with the quantity of the total phenols and flavonoids in *M. limii* plants.

### Comparative Metabolite Profiling by GC-MS and LC-MS Analysis

**Figure 2:**
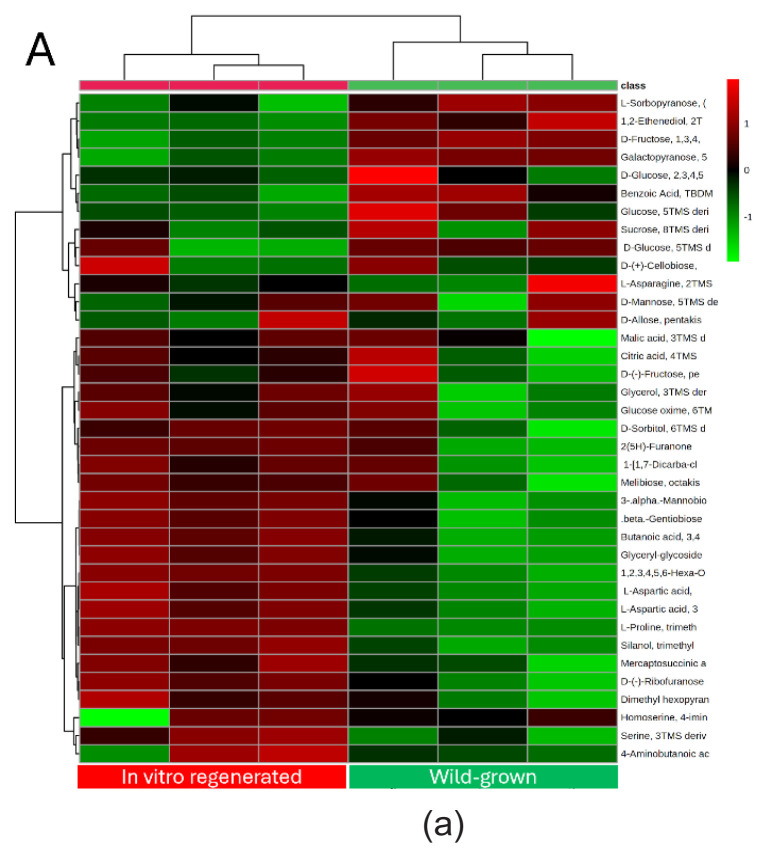

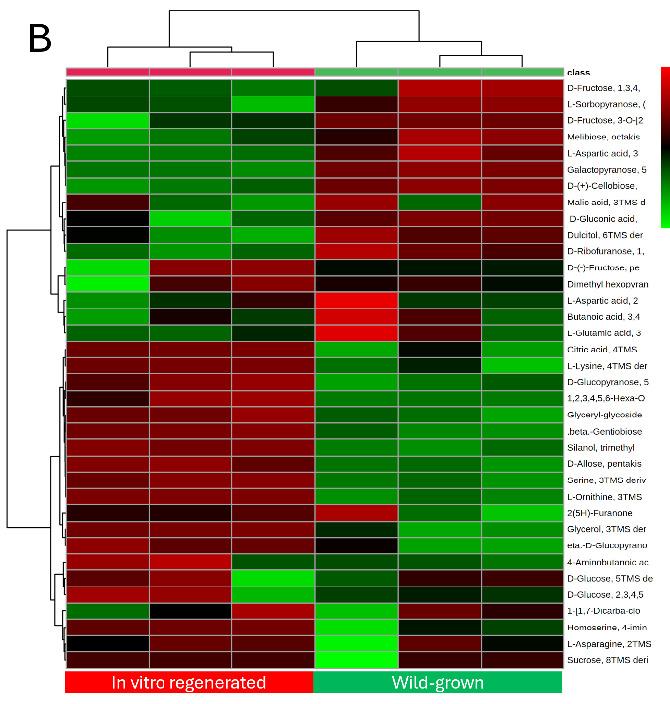

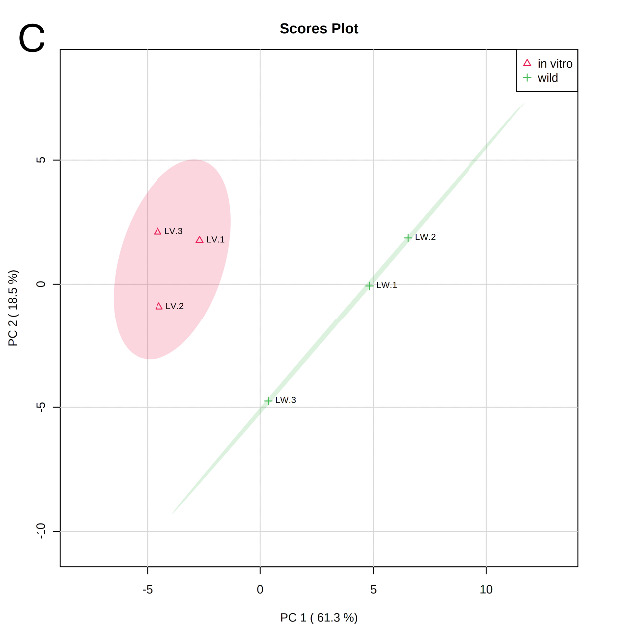

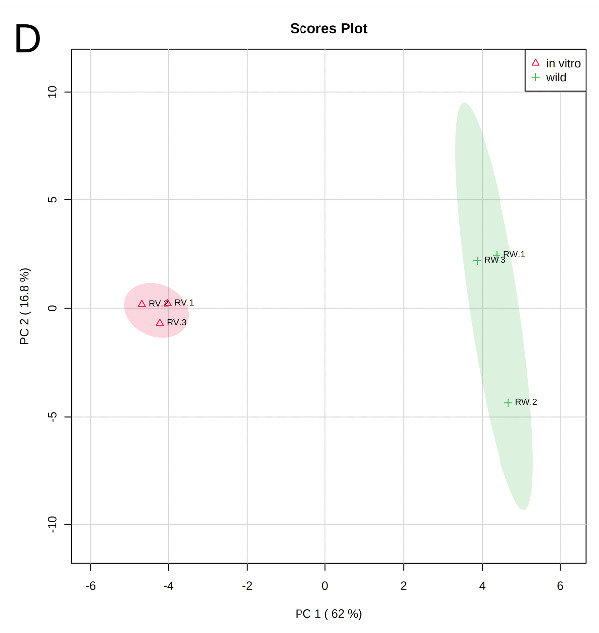
The heatmap analysis from GC-MS analysis showing the distribution of metabolites in (a) leaf and (b) root extracts in both wild and *in vitro* regenerated *M. limii;* PCA score plot of (c) leaf and (d) root extracts of the wild and *in vitro* regenerated *M. limii*.

In GC-MS analysis, a total of 43 metabolites were detected in all samples, with 37 and 36 compounds being tentatively identified in leaf and root parts, respectively (Appendix A). The identified metabolites were grouped as sugar and derivatives, organic acids, amino acids and derivatives, and sugar alcohols. To examine the metabolite variations between the wild and *in vitro* regenerated plants, a multivariate analysis was carried out. The heatmap analysis revealed a high accumulation of sugar derivatives including mannose, D-glucopyranose, fructose, L-sorbopyranose and D-galactopyranose in wild-grown *M. limii* compared to the *in vitro* regenerated plant in both leaf and root extracts [Figs. 2(a) and 2(b)]. Then, the PCA of the leaf samples explained 79.8% of the overall variance of the metabolite profiles, in which the first and second principal components (PCs) separately contributed 61.3% and 18.5%, respectively [Fig. 2(c)]. A similar pattern of separation was observed with the root samples, which revealed 78.8% of the total variation, with 62.0% and 16.8% of the variation as explained by PC1 and PC2, respectively [Fig. 2(d)]. The results revealed that most metabolites in both leaves and roots samples from the wild plant were mostly located in the positive axis, while the *in vitro* propagated plants were mostly displayed in the negative axis, indicating that *M. limii* from different growing environments displayed a significant difference in metabolite composition. To find the features with the power to distinguish metabolites between wild-grown and *in vitro* grown, PLS-DA was further established. Analysis with PLS-DA revealed the contribution of each metabolite in the separation of the groups, and the metabolites were selected based on the VIP score (VIP > 1.0) and *p*-values (*p* < 0.05). Both PLS-DA analyses showed R^2^ = 0.99 and Q^2^ = 0.76 in the leaves part, and R^2^ = 0.99 and Q^2^ = 0.93 for the root part indicating this model has strong predictive power that allowed us to extract metabolite changes from the dataset. A total of 7 and 14 candidate metabolites in the leaves and roots samples of *M. limii* with VIP score of more than 1 (*p* < 0.05) were identified, thus contributed significantly to the distinction between the *in vitro* and wild-grown plants, respectively (Appendix B). In leaves samples, metabolites including L-proline, L-aspartic acids derivatives, butanoic acid and myo-inositol were upregulated in the *in vitro* regenerated plants, and compounds such as galactopyranose, D-fructose and 1,2-ethenediol were downregulated. Meanwhile, the roots extracts revealed that compounds including L-serine, L-ornithine, glyceryl glycoside, glycerol, beta-D-glucopyranose, citric acid, myo-inositol, beta-gentiobiose and L-lysine were upregulated in the *in vitro* regenerated *M. limii*, and compounds such as L-sorbopyranose, D-ribofuranose, galactopyranose, dulcitol and D-cellobiose were downregulated.

**Figure 3:**
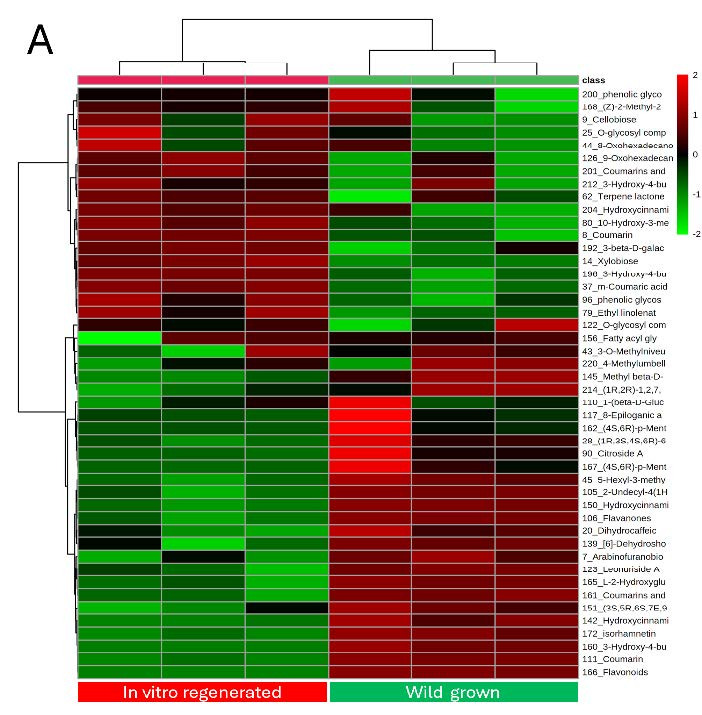

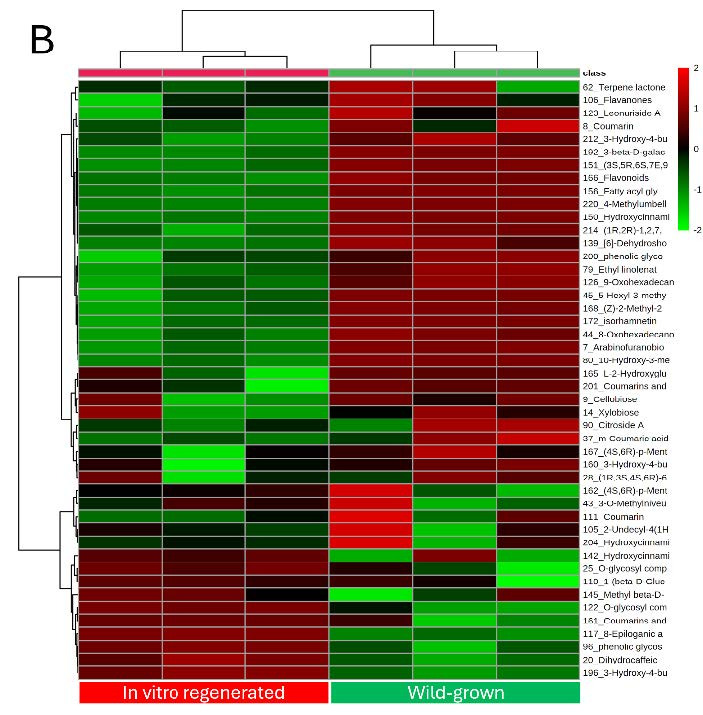

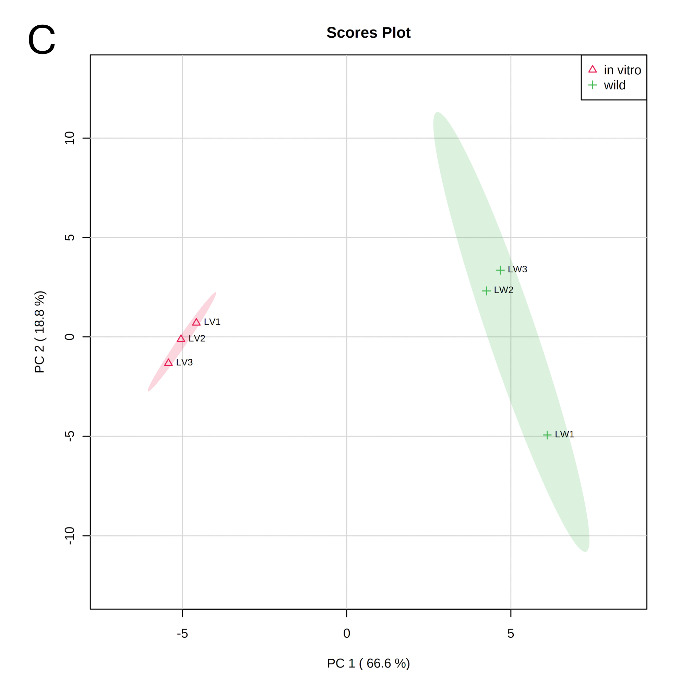

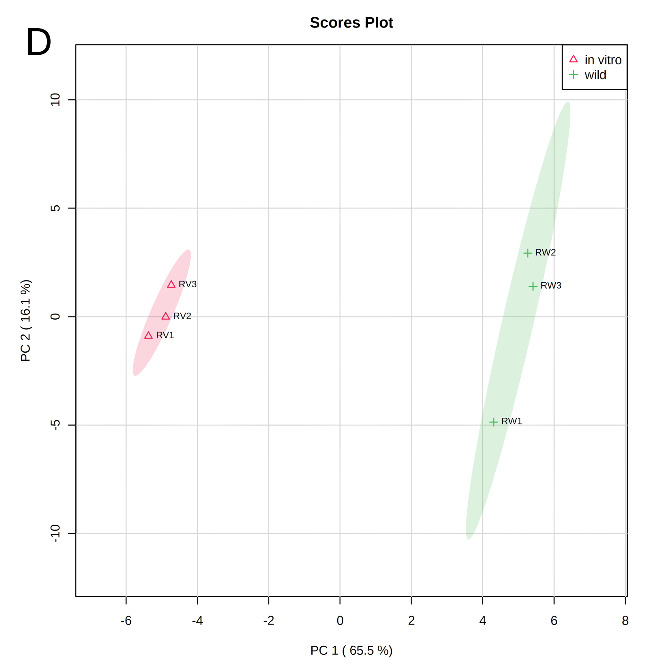
The heatmap analysis from LC-MS/MS analysis showing the distribution of metabolites in (a) leaf and (b) root extracts in both wild and *in vitro* regenerated *M. limii;* PCA score plot of (c) leaf and (d) root extracts of the wild and *in vitro* regenerated *M. limii*.

Analysis with LC-MS/MS revealed a total of 45 metabolites detected in both leaves and roots samples of wild and *in vitro* propagated plants, and were categorised as carbohydrate derivatives, phenolic derivatives, lipids and lipid-like compounds and other metabolites (Appendix C). The heatmap analysis revealed that most metabolites were upregulated in the leaves of the wild plant compared to the *in vitro*-derived leaves [Fig. 3(a)]. A similar pattern was observed in the roots of wild-grown *M. limii* [Fig. 3(b)]. Analysis with PCA showed a substantial difference between two different growing environments of *M. limii* in two principal components explaining 85.4% and 81.6% of the total variability in leaves and root parts, respectively [Figs. 3(c) and 3(d)]. In PLS-DA analysis, both leaves and roots samples of *M. limii* has generated 23 metabolites with VIP values (VIP > 1) and *p*-values (*p* < 0.05), with both PLS-DA analyses showed R^2^ = 0.99 and Q^2^ = 0.97 in the leaves part, and R^2^ = 0.99 and Q^2^ = 0.95 for the root part. Of the 23 metabolites, 15 metabolites from various classes were upregulated in the leaves extracts of wild plants including flavonoids [compound ID (CID): 1, 5, 7], coumarin derivatives (CID: 2, 8), hydroxycinnamic acid derivatives (CID: 4, 9, 20), O-glycosyl compound (CID: 14) phenolic glycosides (CID: 3, 14), lipid derivatives (CID: 19, 23), and two other metabolites including L-2-hydroxyglutaric acid (CID: 11) and a hydroquinolone (CID: 12) (Appendix D). Meanwhile, eight metabolites were downregulated including m-coumaric acid, 3-hydroxy-4-butanolide, a sesquiterpenoids, coumarin, ethyl linoleate, phenolic glycoside, 3-beta-D-galatosyl-sn-glycerol and 9-oxohexadecanoic acid with compound IDs 6, 10, 13, 16, 17, 18, 21 and 22, respectively. For root extracts, the PLS-DA result showed from the 23 significant metabolites detected, 19 metabolites were upregulated in wild plant including coumarin derivatives (CID: 24), hydroxycinnamic acid derivatives (CID: 25, 37), fatty acid derivatives (CID: 26, 30, 35, 36, 40), flavonoids (CID: 31, 33), O-glycosyl compounds (CID: 32, 43, 45), phenolic glycoside (CID: 44), linoleic acid derivative (CID: 39), lipid derivatives (CID: 38, 41), a sesquiterpenoid (CID: 29) and a glycosylglycerol (CID: 27). Only four metabolites were downregulated i.e., a coumarin and three phenolic glycosides with compound IDs 28, 34, 42 and 46, respectively.

## DISCUSSION

### Comparative Phytochemical Contents and Antioxidant Activity in Wild and *In Vitro* Regenerated *M. limii*

Growing conditions significantly influenced the variation of metabolites of *M. limii* plants, whether grown in the wild or through *in vitro* propagation, as revealed in the phytochemical analysis and antioxidant assay. The leaves extract from wild plants exhibited significantly higher level of TPC, TFC, antioxidant capacity, as well as the lowest IC_50_ value for the DPPH assay (6 mg/mL–7 mg/mL) compared to the *in vitro*-derived plants. Previously, the concentrations of plant extracts to inhibit 50% of the DPPH free radical have been reported in *A. formosanus* (4 mg/mL–6 mg/mL), *A. roxburghii* (1 mg/mL–4 mg/mL) and *Anoectochilus burmannicus* (10 mg/mL–12 mg/mL) by Chiang and Lin (2018), Jin *et al*. (2018) and Tangtragoon *et al*. (2023), respectively. In this study, the strong and significant positive correlation between TPC, TFC and antioxidant activity in *M. limii* supports the findings of a previous study by Xie *et al*. (2017). The study suggested that high TPC and TFC levels in *A. roxburghii* contribute to elevated antioxidant activities by DPPH and 2,2-azino-bis-3-ethylbenzothiazoline-6-sulphonic acid (ABTS^+^) scavenging assays. These variations in responses were expected and may be attributed to the physical and chemical environments in which the plants grow. In natural ecosystems, factors including climate, soil and geographic location, cultural practices can have a major impact on increasing or decreasing the quantity and quality of plant performance (Abu-Qaoud *et al*. 2018; Zargoosh *et al*. 2019). The current finding was also in line with Li *et al*. (2017), that the flavonoids and polysaccharide contents in *A. roxburghii* were significantly higher in the soil-cultivated seedlings than in the tissue-cultured seedlings. In other studies, the phenolic and flavonoid contents, as well as their antioxidant activity in *A. formosanus* were also influenced by growing medium (Nguyen *et al*. 2018) and drying treatments (Chiang & Lin 2018).

### Mass Spectrometry-based Metabolite Profiling of *M. limii*

Untargeted metabolomics has been extensively utilised to compare the varieties of metabolic composition in samples that reflect the dynamic responses to physiological change or developmental stimuli (Perez *et al*. 2019). In this study, the combination of GC-MS and LC-MS/MS approaches have successfully identified the metabolites in *M. limii* plants including sugar alcohol, sugar derivatives, organic acids, amino acids, carbohydrate derivatives, phenolic derivatives, and lipid and lipid-like compounds. Previously, bioactive compounds such as amino acids, organic acids, polysaccharides, and flavonoids were reported to contribute to the therapeutic effects in jewel orchids including *A. elatus*, *A. roxburghii*, *A. formosanus* and *L. discolor* (Wu *et al*. 2020; Ye *et al*. 2020). The current finding revealed that sugar derivatives constitute the major plant metabolites in *M. limii* by GC-MS analysis. Sugars are primary photosynthetic products that are involved in a wide variety of metabolic pathways, growth, and developmental processes in plants (Yoon *et al*. 2021). Previously, it was reported that glucose and galactose constitute the primary structural component in the polysaccharides of *A. roxburghii* (Zhang *et al*. 2020). Then, Wu *et al*. (2021) added that the polysaccharides in *A. roxburghii* are rod-like aggregates, with no branching, uniform size, and are primarily made up of arabinose, glucose, rhamnose, mannose, xylose, and galactose in the molar ratio of 0.28: 1.93: 2.06: 2.40: 1.00: 6.43. Later, another study revealed that the combination of glucose and galactose compounds at 75.2% and 14.5%, respectively, with α-type glycosidic chains, are the main structure of polysaccharides in *A. roxburghii* (Jin *et al*. 2022). Polysaccharides have been reported to be the main active ingredient in *Anoectochilus* jewel orchids with various bioactivities including antioxidant (Nguyen *et al*. 2023), anticancer (Chung *et al*. 2021), antinociceptive effect (Shi *et al*. 2023), and hepatoprotective effects (Wu *et al*. 2022).

Analysis with LC-MS/MS revealed the presence of phenolic derivatives as the major metabolites in both leaves and roots of *M. limii* plants. Among them, a phenolic glycoside compound, tentatively identified as kinsenoside (C_10_H_6_O_8_), was detected at higher levels in leaf and root extract of wild *M. limii* plants. Kinsenoside which was first isolated from *Anoectochilus koshunensis* (Ito *et al*. 1993), was also isolated from *A. formosanus* (Du *et al*. 2001) and *A. roxburghii* (Liu *et al*. 2014). It has been reported that kinsenoside is the main active compound responsible for promoting these jewel orchids as the “King of Medicine” (Qi *et al*. 2018). The benefits of kinsenoside in jewel orchids includes its anti-hyperliposis effect (Du *et al*. 2001), hepatoprotective activity (Hsieh *et al*. 2011) treating diabetic vascular disease (Liu *et al*. 2013), potential as an antidiabetic drug candidate (Rehman *et al*. 2015), as well as the anti-inflammatory property (Karinchai *et al*. 2021). However, the tentatively identified kinsenoside detected in *M. limii* plants from this study requires additional investigation to confirm its identity. Protocol to extract and purify the kinsenoside compound has been established in *A. roxburghii* with deep eutectic solvent by column chromatography extraction (Yuan *et al*. 2022). Other phenolic compounds that were also significant in the wild-grown *M. limii* include flavonoids, coumarin derivatives, hydrocinnamic acid glycosides, and flavonones. Previously, flavonoid glycosides were isolated from *A. roxburghii* including quercetin-7-O-β-D-[6″-O-(trans-feruloyl)]-glucopyranoside (He *et al*. 2006) and roxburoside (Liu *et al*. 2014). Recently, two new flavone glycosides, isorhamnetin-3-O-α-L-rhamnosyl-(1→6)-β-D-glucopyranose-(1→3)-β-D-glucopyranoside and kaempferol-7-O-β-D-glucopyranosyl-(1→3)-β-D-glucopyranoside were isolated from ethanol extract of *A. roxburghii* (Bin *et al*. 2023). The flavonoid glycosides isolated from jewel orchids have been reported to have anti-inflammatory activity (Hoi *et al*. 2016), antioxidant activity (He *et al*. 2006; Liu *et al*. 2014), as well as anti-aging properties (Wang *et al*. 2020).

### *In Vitro* Regenerated *M. limii* Offer a Viable Alternative to Wild-grown Plants

Multivariate analysis revealed that the metabolites regulation in wild and *in vitro* propagated *M. limii* plants gave varied responses. The purpose of utilising *in vitro* propagated plants in this study was to alleviate the scarcity of wild resource shortage due to medicinal and ornamental purposes. Currently, the tissue-cultured jewel orchids including *A. roxburghii* and *A. formosanus* have been used commercially in the pharmacology industry as the source of flavonoids as well as polysaccharides (Wu *et al*. 2021; Nguyen *et al*. 2023). For *M. limii* plants, protocol to regenerate this plant by *in vitro* technique has been established previously (David *et al*. 2022). Even though the phytochemical content, antioxidant activity as well as some metabolites in the leaf and root extracts of the *in vitro* regenerated *M. limii* plants were slightly lower compared to the wild-grown plants, previous studies have showed that the manipulation of various factors could increase the bioactive compounds in the tissue-cultured jewel orchids. For instance, the addition of abiotic elicitors salicylic acid and methyl jasmonate to culture medium has increased the polysaccharide and kinsenoside contents in rhizome culture of *A. roxburghii* (Luo *et al*. 2018). In another study, treatment with LED lighting technology has positively impacted the growth along with the content of soluble sugar, polysaccharides and total flavonoids in *A. roxburghii* (Wang *et al*. 2018; Gam *et al*. 2020) and *A. burmannicus* (Tangtragoon *et al*. 2023).

## CONCLUSION

For the first time, the metabolite composition of *M. limii* jewel orchids grown both in the wild and through *in vitro* cultivation has been reported. Different growing environments significantly influenced the metabolite variations in *M. limii* plants. The mass spectrometry-based approach revealed a significant level of sugar derivatives and phenolic compounds in *M. limii* plants, which were previously reported to have various medicinal benefits. However, further investigation into the therapeutic potential of this plant extract is encouraged to explore its natural bioactive compounds, which could be valuable in drug discovery. Hence, the utilisation of *in vitro* regenerated *M. limii* plants for future research is recommended for bioactive compound production, as well as for mass propagation to support conservation efforts for this indigenous plant.

## APPENDICES

### Appendix A Putatively identified compounds in leaf and root extracts of both wild and *in vitro* regenerated *M. limii* based on peak area (%) using GC-MS analysis

[Table t1-tlsr_35-3-23]

**Table t1-tlsr_35-3-23:**

No.	m/z	RT(min)	Compound name	Peak area (%)			

				Leaves		Roots	
	
				Wild	*In vitro*	Wild	*In vitro*
Organic acids							

1	55	5.92	2(5H)-furanone	0.89	4.65	2.41	2.31
2	147	20.53	Butanoic acid, 3,4-bis[(TMS) oxy]-, TMS ester	0.24	0.69	0.23	0.17
3	233	21.77	Malic acid, (3TMS)	1.97	2.19	1.07	0.10
4	209	22.65	4-aminobutanoic acid,(3TMS)	0.36	0.83	0.27	0.44
5	274	29.58	Citric acid, (4TMS)	0.38	0.35	0.02	0.19
6	179	15.92	Benzoic acid, (TBDMS)	0.28	0.07	ND	ND
7	147	19.71	Mercaptosuccinic acid, (3TMS)	0.12	0.24	ND	ND

Sugar alcohols							

8	299	16.60	Silanol, TMS-, phosphate(3:1)	2.01	7.25	0.81	9.13
9	205	16.76	Glycerol, (3TMS)	0.53	0.57	0.01	0.31
10	73	7.97	1,2-ethenediol, (2TMS)	0.96	0.12	0.00	0.00
11	73	38.36	Myo-inositol (6TMS)	0.10	0.74	0.12	0.43

Amino acid and derivatives							

12	70	13.93	L-proline, TMS ester	Trace	1.33	ND	ND
13	204	18.82	Serine, (TMS)	0.05	0.31	0.23	0.40
14	160	20.28	L-aspartic acid, (2TMS)	0.36	3.52	0.74	0.42
15	73	22.48	L-aspartic acid, (3TMS)	0.14	0.56	7.67	4.98
16	44	24.03	L-asparagine, (2TMS)	1.80	0.53	0.22	0.23
17	70	24.45	L-ornithine, (3TMS)	ND	ND	0.01	1.64
18	246	24.54	L-glutamic acid, (3TMS)	ND	ND	0.62	0.46
19	116	25.55	Homoserine, 4-imino-N,O-bis(TMS)-, TMS ester	0.08	0.09	0.18	0.72
20	156	33.71	L-lysine, (4TMS)	ND	ND	0.01	0.18

Sugars and derivatives							

21	103	22.86	Fructose, MEOX-5TMS-1	0.27	0.04	0.33	0.08
22	204	29.19	Galactopyranose, (5TMS)	3.26	0.97	5.19	1.37
23	204	30.65	1-[1,7-dicarba-closo-dodecaboran-(12)-1-yl]-D-glucopyranose, (4TMS)	5.05	7.33	1.95	1.72
24	353	31.33	L-sorbopyranose, (1S,2R,3S)-, (5TMS)	0.03	0.01	0.96	0.02
25	217	31.63	Fructose, MEOX-5TMS-2	4.07	3.55	1.81	2.63
26	157	31.91	D-sorbitol, (6TMS)	0.16	0.38	0.25	0.08
27	204	32.57	D-mannose (5TMS)	32.63	27.01	22.55	20.22
28	160	32.74	D-glucose, 2,3,4,5,6-pentakis-O-(TMS)-,O-methyloxime	1.84	1.06	2.04	2.11
29	160	33.49	D-allose, pentakis(TMS)ether,methyloxime (syn)	0.61	0.64	0.33	0.45
30	205	33.54	Glucose oxime, (6TMS)	2.32	2.37	ND	ND
31	215	33.54	D-ribofuranose, 1,2,3,5-tetrakis-O-(TMS)-	Trace	0.07	0.17	0.02
32	204	36.00	D-glucopyranose, (5TMS)	27.60	10.14	21.74	29.81
33	407	36.62	D-gluconic acid, (6TMS)	ND	ND	0.09	0.02
34	204	37.11	beta-D-glucopyranose,(5TMS)	ND	ND	0.05	0.13
35	204	42.17	Glyceryl-glycoside TMS ether	0.60	1.78	0.07	0.51
36	290	43.45	Glucose, (5TMS)	0.62	0.08	ND	ND
37	204	43.88	Melibiose, octakis (TMS)-	0.79	0.91	12.98	7.64
38	217	44.23	Dimethyl hexopyranosiduronate, alpha-D-, (3TMS)	4.24	11.45	3.04	2.49
39	73	46.09	Sucrose, (8TMS)	1.16	0.41	2.68	5.54
40	268	46.41	D-fructose, 3-O-[2,3,4,6-tetrakis-O-(TMS)-alpha-D-glucopyranosyl]-1,4,5,6-tetrakis-O-(TMS)	ND	ND	1.07	0.19
41	173	47.46	3-alpha-mannobiose, octakis(TMS) ether, methyloxime (isomer 1)	0.01	0.04	ND	ND
42	204	47.60	D-(+)-cellobiose, (isomer 2),(8TMS)	1.84	1.94	8.09	2.79
43	160	47.88	Beta-gentiobiose, octakis(TMS) ether, methyloxime (isomer 1)	2.62	5.79	0.02	0.10

*Notes*: RT = retention time; TMS = trimethylsily; MEOX = methyloxime, ND = not detected

### Appendix B PLS-DA modelling of discriminant putatively identified compounds by variable importance in the projection (VIP) in leaves and root extracts of *M. limii* from wild and *in vitro* grown by GC-MS analysis

[Table t2-tlsr_35-3-23]

**Table t2-tlsr_35-3-23:**

Name	RT (min)	m/z	*p-*value	VIP	Log(FC)
Leaves					

L-proline	13.93	147	1.86E-05	1.3758	12.9290
Myo-inositol	38.36	73	0.002608	1.3226	3.0860
L-aspartic acid	20.30	160	0.004752	1.3022	3.5305
Butanoic acid	20.53	233	0.010067	1.2659	1.7767
D-fructose	31.65	217	0.000736	1.3499	−2.4557
Galactopyranose	29.19	204	0.001080	1.3434	−1.5168
1,2-ethenediol	7.97	73	0.007372	1.2827	−2.7170

Roots					

L-ornithine	24.45	70	6.48E-05	1.2639	7.3794
Serine	18.82	204	0.000167	1.2588	1.0381
Beta-gentiobiose(isomer 1)	47.88	160	0.000202	1.2575	2.3564
Glyceryl-glycoside	42.17	204	0.001194	1.2362	3.0640
Myo-inositol	38.35	217	0.003656	1.2089	2.1116
Glycerol	16.76	205	0.004756	1.2000	5.2229
L-lysine	33.71	156	0.011792	1.1577	4.7594
Citric acid	29.58	274	0.013194	1.1510	3.4267
Beta-D-glucopyranose	37.11	204	0.023902	1.1081	1.7144
Galactopyranose	29.19	204	3.70E-05	1.2660	−1.6558
D-(+)-cellobiose,(isomer 2)	47.60	204	0.000354	1.2527	−1.2724
D-ribofuranose	33.54	215	0.004229	1.2041	−3.1764
L-sorbopyranose	31.33	353	0.013398	1.1501	−5.7351
Dulcitol	31.91	157	0.022339	1.1137	−1.3054

*Note*: The list of VIP scores is provided together with log fold-change (LogFC) values

### Appendix C Putatively annotated compounds in leaf and root parts of *M. limii* by LC-QTOF-MS/MS analysis

[Table t3-tlsr_35-3-23]

**Table t3-tlsr_35-3-23:**

RT (min)	m/z	Adducts	Exact mass	m/z error (ppm)	Molecular formula	Putative annotation	Peak area (%)			

							Root		Leaf	
	
							Wild	*In vitro*	Wild	*In vitro*
Carbohydrate derivatives										

0.93	365	[M+Na] +	342	2	C12H22O11	Cellobiose	9.70	20.44	21.04	22.95
0.98	305	[M+Na] +	282	0	C10H18O9	Xylobiose	3.69	10.67	0.28	0.27
1.34	305	[M+Na] +	282	0	C10H18O9	Arabinofuranobiose	13.28	12.03	0.64	0.02
1.36	217	[M+Na] +	194	1	C7H14O6	Methyl beta-D-glucopyranoside	14.70	0.01	8.38	15.61
12.57	453	[M+Na] +	430	–	C20H30O10	O-glycosyl compounds*	1.64	4.97	0.08	0.47
12.79	453	[M+Na] +	430	–	C20H30O10	O-glycosyl compounds*	0.50	0.56	0.03	0.42
Lipids and lipid-like molecules										
2.22	255	[M+H] +	254	5	C9H18O8	3-beta-D-galactosyl-sn-glycerol	0.01	0.11	3.16	0.06
2.86	261	[M+H] +	260	0	C16H20O3	10-hydroxy-3-methoxy-1,3,5,7-cadinatetraen-9-one	0.03	0.64	0.43	0.01
3.57	315	[M+Na] +	292	–	C12H20O8	Fatty acyl glycosides	0.01	0.01	0.30	0.01
4.74	287	[M+Na] +	264	1	C11H20O7	(Z)-2-methyl-2-butene-1,4-diol 4-O-beta-D-glucopyranoside	0.14	0.16	0.10	0.06
5.72	369	[M+Na] +	346	1	C16H26O8	(1R,3S,4S,6R)-6,9-dihydroxyfenchone 6-O-b-D-glucoside	2.30	2.08	0.01	0.02
6.01	355	[M+Na] +	330	2	C16H28O7	(4S,6R)-p-mentha-1,8-diene-6,7-diol 7-glucoside	0.67	0.09	0.10	0.11
6.3	329	[M+Na] +	306	2	C14H26O7	1-(beta-D-glucopyranosyloxy)-3-octanone	0.15	0.18	0.54	2.40
6.63	409	[M+Na] +	386	0	C19H30O8	Citroside A	1.65	0.53	0.86	0.02
6.74	353	[M+Na] +	330	0	C16H26O7	(4S,6R)-p-mentha-1,8-diene-6,7-diol 7-glucoside	0.35	0.17	0.01	0.00
7.45	409	[M+H] +	408	3	C21H28O8	3-O-methylniveusin A	0.75	1.21	0.03	0.05
7.97	323	[M+H] +	322	3	C20H34O3	5-hexyl-3-methyl-2-furannonanoic acid	0.72	0.74	0.84	0.04
8.19	307	[M+H] +	306	4	C20H34O2	Ethyl linolenate	0.06	0.38	6.60	0.04
8.56	413	[M+H] +	390	0	C19H34O8	(3S,5R,6S,7E,9x)-7-megastigmene-3,6,9-triol 9-glucoside	0.03	0.02	1.48	0.02
12.79	415	[M+Na] +	392	–	C22H32O6	Terpene lactone*	0.04	0.22	0.71	0.03
12.81	293	[M+Na] +	270	1	C16H30O3	8-oxohexadecanoic acid	0.99	2.18	0.16	0.03
12.83	191	[M+Na] +	168	3	C11H20O	(1R,2R)-1,2,7,7-tetramethylbicyclo[2.2.1]heptan-2-ol	0.49	0.01	0.41	0.02
13.01	293	[M+Na] +	270	2	C16H30O3	9-oxohexadecanoic acid	0.00	0.04	0.87	0.16

Phenolic derivatives										

0.92	381	[M+Na] +	358	0	C15H18O10	Dihydrocaffeic acid 3-O-glucuronide	7.56	9.67	6.26	20.51
1.02	287	[M+Na] +	264	0	C10H16O8	Kinsenoside	15.78	0.00	23.47	15.05
2.16	265	[M+H] +	264	0	C10H16O8	Kinsenoside	0.11	11.19	0.01	4.02
4.78	333	[M+H] +	332	–	C14H20O9	Phenolic glycosides*	0.67	0.03	3.45	3.70
1.99	165	[M+H] +	164	2	C9H8O3	m-coumaric acid	0.09	4.46	0.08	0.00
2.17	165	[M+H] +	164	–	C9H8O3	Hydroxycinnamic acids andderivatives*	0.23	0.03	0.24	0.81
3.65	331	[M+Na] +	308	5	C15H16O7	4-methylumbelliferyl beta-D-xylopyranoside	0.01	0.00	2.35	0.05
4.74	313	[M+H] +	312	–	C18H16O8	Hydroxycinnamic acid glycosides*	0.10	0.64	0.24	0.05
5.13	177	[M+H] +	176	–	C10H8O3	Coumarins and derivatives*	0.01	0.07	0.25	0.12
5.88	349	[M+Na] +	326	–	C15H18O8	Hydroxycinnamic acid glycosides*	0.82	0.07	5.19	0.01
6.85	147	[M+H] +	146	3	C9H6O2	Coumarin	5.58	0.06	0.03	0.00
6.85	433	[M+H] +	433	–	C21H21O10	Flavanones*	9.46	0.01	0.03	0.01
6.99	297	[M+Na] +	274	2	C17H22O3	[6]-Dehydroshogaol	0.70	0.10	0.17	0.04
7.13	147	[M+H] +	146	–	C9H6O2	Coumarins and derivatives*	3.66	0.04	0.47	5.64
7.36	147	[M+H] +	146	1	C9H6O2	Coumarin	0.49	15.74	0.28	0.30
7.5	317	[M+H] +	316	–	C16H12O7	Flavonoids*	0.50	0.00	0.50	0.06
8.09	317	[M+H] +	316	2	C16H12O7	Isorhamnetin	0.24	0.01	0.87	0.00

Others										

2.33	171	[M+Na] +	148	4	C5H8O5	L-2-hydroxyglutaric acid	0.91	0.01	7.50	3.47
5.1	322	[M+Na] +	299	4	C20H29NO	2-undecyl-4(1H)-quinolinone	0.96	0.01	0.07	0.02

*Note*:

* Annotation level: compound was annotated at the level of a specific class with the molecular formula

### Appendix D PLS-DA modelling of discriminant putatively identified compounds by variable importance in the projection (VIP) in leaves and root extracts of *M. limii* from wild and *in vitro* grown by LC-QTOF-MS/MS analysis

[Table t4-tlsr_35-3-23]

**Table t4-tlsr_35-3-23:**

Compound ID (CID)	Name	*p*-value	VIP	log(FC)	Class
Leaves					

1	Flavonoids	1.50E^-06^	1.2311	2.4135	Flavonoids
2	Coumarin	1.88E^-05^	1.2280	7.3533	Coumarins and derivatives
3	3-hydroxy-4-butanolide	9.17E^-05^	1.2227	2.5048	Phenolic glycoside
4	Hydroxycinnamic acid glycosides	0.00013708	1.2205	4.4405	Hydroxycinnamic acid glycosides
5	Isorhamnetin	0.00020344	1.2180	5.6265	Flavonoids
6	m-*C*oumaric acid	0.00040242	1.2121	−4.7491	Hydroxycinnamic acids and derivatives
7	Flavanones	0.00055423	1.2086	10.589	Flavanones
8	Coumarins and derivatives	0.00078089	1.2041	7.1654	Coumarins and derivatives
9	Hydroxycinnamic acids and derivatives	0.00090706	1.2019	3.6967	Hydroxycinnamic acid glycosides
10	3-hydroxy-4-butanolide	0.00157340	1.1922	−5.7666	Phenolic glycoside
11	L-2-hydroxyglutaric acid	0.00166810	1.1910	7.2058	L-2-hydroxyglutaric acid
12	2-undecyl-4(1H)-quinolinone	0.00189960	1.1882	7.3741	Hydroquinolones
13	10-hydroxy-3-methoxy-1,3,5,7-cadinatetraen-9-one	0.00334300	1.1737	−3.7560	Sesquiterpenoids
14	Methyl beta-D-glucopyranoside	0.00459530	1.1635	10.9780	O-glycosyl compounds
15	Leonuriside A	0.00482870	1.1618	5.0739	Phenolic glycosides
16	Coumarin	0.00500740	1.1604	−4.0750	Coumarins and derivatives
17	Ethyl linolenate	0.01236800	1.1187	−1.9816	Lineolic acids and derivatives
18	Phenolic glycosides	0.02086600	1.0840	−2.3903	Phenolic glycosides
19	(3S,5R,6S,7E,9x)-7-megastigmene-3,6,9-triol 9-glucoside	0.02354600	1.0745	1.2504	Fatty acyl glycosides
20	[6]-dehydroshogaol	0.02432500	1.0719	3.5146	Hydroxycinnamic acids and derivatives
21	3-beta-D-galactosyl-sn-glycerol	0.03309100	1.0445	−3.5702	Glycosylglycerols
22	9-Oxohexadecanoic acid	0.03645500	1.0349	−2.0676	Long-chain fatty alcohols
23	(1R,2R)-1,2,7,7-tetramethylbicyclo[2.2.1]heptan-2-ol	0.04292800	1.0175	7.1792	Prenol lipids

Roots					

24	4-methylumbelliferyl beta-D-xylopyranoside	2.78E-^07^	1.2379	6.2848	Coumarins and derivatives
25	Hydroxycinnamic acid glycosides	1.77E-^06^	1.2371	9.3271	Hydroxycinnamic acid glycosides
26	Fatty acyl glycosides	2.20E-^05^	1.2337	5.9711	Fatty acyl glycosides
27	3-beta-D-galactosyl-sn-glycerol	2.64E-^05^	1.2333	6.3811	Glycosylglycerols
28	8-epiloganic acid	4.03E-^05^	1.2320	−7.3899	Coumarins and derivatives
29	10-hydroxy-3-methoxy-1,3,5,7-cadinatetraen-9-one	4.79E-^05^	1.2315	6.4630	Sesquiterpenoids
30	(3S,5R,6S,7E,9x)-7-megastigmene-3,6,9-triol 9-glucoside	5.21E-^05^	1.2312	6.8259	Fatty acyl glycosides
31	Flavonoids	6.15E-^05^	1.2305	3.6450	Flavonoids
32	Arabinofuranobiose	7.51E-^05^	1.2297	5.9245	O-glycosyl compounds
33	isorhamnetin	0.00040539	1.2180	8.3609	Flavonoids
34	3-hydroxy-4-butanolide	0.00043247	1.2174	−7.9149	O-glycosyl compounds
35	8-oxohexadecanoic acid	0.00058948	1.2138	3.0157	Long-chain fatty acids
36	(Z)-2-methyl-2-butene-1,4-diol 4-O-beta-D-glucopyranoside	0.00067113	1.2122	1.3977	Fatty acyl glycosides
37	[6]-dehydroshogaol	0.00090765	1.2079	2.6926	Hydroxycinnamic acids and derivatives
38	(1R,2R)-1,2,7,7-tetramethylbicyclo[2.2.1]heptan-2-ol	0.00135910	1.2010	5.2595	Prenol lipids
39	Ethyl linolenate	0.00177990	1.1955	7.9373	Lineolic acids and derivatives
40	9-oxohexadecanoic acid	0.00207050	1.1922	3.0406	Long-chain fatty alcohols
41	5-hexyl-3-methyl-2-furannonanoic acid	0.00265820	1.1860	4.8434	Lipids and lipid-like molecules
42	Dihydrocaffeic acid 3-O-glucuronide	0.00286770	1.1839	−1.1068	Phenolic glycosides
43	3-hydroxy-4-butanolide	0.00442370	1.1706	3.3511	O-glycosyl compounds
44	phenolic glycosides	0.00495660	1.1666	4.2412	Phenolic glycosides
45	O-glycosyl compounds	0.01068300	1.1324	3.0266	O-glycosyl compounds
46	phenolic glycosides	0.02145400	1.0872	−3.1993	Phenolic glycosides

*Note*: The list of VIP scores is provided together with log fold-change (LogFC) values
